# Eosinophils enhance WNT-5a and TGF-β_1_ genes expression in airway smooth muscle cells and promote their proliferation by increased extracellular matrix proteins production in asthma

**DOI:** 10.1186/s12890-016-0254-9

**Published:** 2016-06-13

**Authors:** Andrius Januskevicius, Simona Vaitkiene, Reinoud Gosens, Ieva Janulaityte, Deimante Hoppenot, Raimundas Sakalauskas, Kestutis Malakauskas

**Affiliations:** Laboratory of Pulmonology, Department of Pulmonology and Immunology, Lithuanian University of Health Sciences, Eiveniu str. 2, Kaunas, LT-50009 Lithuania; Department of Molecular Pharmacology, University of Groningen, Antonius Deusinglaan 1, 9713 AV Groningen, The Netherlands; Department of Pulmonology and Immunology, Lithuanian University of Health Sciences, Eiveniu str. 2, Kaunas, LT-50009 Lithuania

**Keywords:** Eosinophils, Airway smooth muscle cells, Adhesion, Proliferation, WNT-5a, TGF-β_1_, Fibronectin, Collagen, Asthma

## Abstract

**Background:**

Recent studies have suggested that eosinophils may have a direct effect on airway smooth muscle cells (ASMC), causing their proliferation in patients with asthma, but the precise mechanism of the interaction between these cells remains unknown. We propose that changes in Wnt signaling activity and extracellular matrix (ECM) production may help explain these findings. Therefore, the aim of this study was to investigate the effect of eosinophils from asthmatic and non-asthmatic subjects on Wnt-5a, transforming growth factor β_1_ (TGF-β_1_), and ECM protein (fibronectin and collagen) gene expression and ASMC proliferation.

**Methods:**

A total of 18 subjects were involved in the study: 8 steroid-free asthma patients and 10 healthy subjects. Peripheral blood eosinophils were isolated using centrifugation and magnetic separation. An individual co-culture of eosinophils with human ASMC was prepared for each study subject. Adhesion of eosinophils to ASMC (evaluated by assaying eosinophil peroxidase activity) was determined following various incubation periods (30, 45, 60, 120, and 240 min). The expression of Wnt-5a, TGF-β_1_, and ECM protein genes in ASMC was measured using quantitative real-time polymerase chain reaction (PCR) after 24 h of co-culture. Proliferation of ASMC was measured using the Alamar blue method after 48 h and 72 h of co-culture with eosinophils.

**Results:**

Eosinophils from asthmatic subjects demonstrated increased adhesion to ASMC compared with eosinophils from healthy subjects (*p* < 0.05) in vitro. The expression of Wnt-5a, TGF-β_1_, collagen, and fibronectin genes in ASMC was significantly higher after 24 h of co-culture with eosinophils from asthmatic subjects, while co-culture of ASMC with eosinophils from healthy subjects increased only TGF-β_1_ and fibronectin gene expression. ASMC proliferation was augmented after co-culture with eosinophils from asthma patients compared with co-culture with eosinophils from healthy subjects (*p* < 0.05).

**Conclusions:**

Eosinophils enhance Wnt-5a, TGF-β_1_, fibronectin, and collagen gene expression in ASMC and promote proliferation of these cells in asthma.

**Trial registration:**

ClinicalTrials.gov Identifier: NCT02648074.

## Background

Asthma is a chronic inflammatory disorder of the airways in which many cells and cellular elements play critical roles. It is characterized by chronic inflammation, which is associated with airway hyperresponsiveness that leads to recurrent episodes of coughing, wheezing, breathlessness, and chest tightness [1]. Eosinophils are circulating granulocytes produced in the bone marrow that can be recruited to sites of immunological or inflammatory responses [2]. In response to interleukin 5 (IL-5), eosinophils attach to airway endothelium and, in the presence of cytokines CCL11 and CCL5, migrate to the submucosa and lamina propria, where they can make contact with airway smooth muscle cells (ASMC) in vivo [3]. Recruitment of eosinophils leads to airway remodeling owing to secreted eosinophil cytokines and chemokines. Airway remodeling encompasses cellular and structural changes in the airway [4]. These changes include subepithelial fibrosis, smooth muscle hypertrophy/hyperplasia [5, 6], epithelial cell mucus metaplasia, greater extracellular matrix (ECM) production [7], and angiogenesis [8, 9]. Increased airway smooth muscle mass is a pathological feature in individuals with asthma [10].

Halwani et al. showed that integrins of eosinophils bind to ASMC adhesion molecules and that this interaction is required for the release of mediators leading to ASMC proliferation in asthma [11]. Other studies suggest that eosinophils induce ECM protein expression in ASMC [12], which enhances ASMC proliferation [13]. However, precisely how eosinophils cause ASMC proliferation in asthma is unknown. One possible mechanism is altered noncanonical Wnt signaling in ASMC after direct contact with eosinophils.

Wnt signaling plays a critical role in the development of multicellular organisms and the maintenance of adult tissue homeostasis [14, 15]. Wnt-5a is a member of a family of 19 cysteine-rich secreted glycoproteins; it activates noncanonical Wnt signaling [16] through c-Jun N-terminal kinase-dependent or Ca^2+^-dependent pathways [15]. There is crosstalk between Wnt-5a ligand expression and transforming growth factor β_1_ (TGF-β_1_) [12]. TGF-β_1_ is one of the major players in determining the structural and functional abnormalities of airway smooth muscle in asthma [9]. The interaction between TGF-β_1_ and Wnt-5a is associated with various pathophysiological functions in asthma [12]. Expression and activation of TGF-β_1_ leads to airway remodeling in asthmatic lungs [17] and may induce Wnt signaling in human ASMC [18].

We hypothesized that eosinophils promote ASMC proliferation through elevated ECM protein (fibronectin and collagen) production induced by altered Wnt-5a and TGF-β_1_ expression in asthma. To test this hypothesis, we used co-cultures of peripheral blood eosinophils isolated from asthma patients or healthy individuals and healthy immortalized human ASMC.

## Methods

### Subjects

A total of 18 nonsmoking adults (men and women) were examined: 8 patients with persistent mild to moderate asthma defined according the GINA criteria and 10 healthy subjects who comprised the control group. The patients were recruited from the Department of Pulmonology and Immunology, Hospital of the Lithuanian University of Health Sciences, Kaunas. The study protocol was approved by the Regional Biomedical Research Ethics Committee of the Lithuanian University of Health Sciences (BE-2-13), and each participant gave his/her informed written consent.

Patients with asthma had a clinical history of the disease for ≥1 year, current symptoms, airway hyperresponsiveness and positive skin prick test (≥3 mm) in response to house dust mites *Dermatophagoides pteronyssinus* (*D. pteronyssinus*) or *Dermatophagoides farinae* (*D. farinae*). All patients were not using inhaled, nasal or oral steroids at least 1 month before visits, short-acting β2 agonists at least 12 h and long-acting β2 agonists at least 48 h prior the lung function test, antihistamines and antileukotrienes – 7 days before skin prick test and prior the lung function test. None of the patients had a history of smoking. Baseline forced expiratory volume in one second (FEV1) was more than 70 % of the predicted value in all patients. All healthy subjects were nonsmokers, without symptoms of rhinitis or asthma, with normal findings of spirometry and methacholine challenge, and all showed negative results of skin prick test (Table 1).

**Table 1:** Demographic, clinical and laboratory data of study subjects

Characteristics	Asthma patients *n* = 8	Healthy subjects *n* = 10
Age (years)	32.0 ± 3.4	26.2 ± 0.9
Gender (M/F), n	3/5	3/7
FEV_1_ (L)	3.92 ± 0.23	3.68 ± 0.21
FEV_1_ (% of predicted)	109.6 ± 3.6	100.7 ± 2.7
Wheal diameter induced by allergen (mm)	4.8 ± 0.6	0
PD_20_ (mg)	0.11^a^	–
Blood eosinophil count (x 10^9^/L)	0.23 ± 0.05*	0.12 ± 0.01
Sputum eosinophil count (× 10^9^/L)	0.09 ± 0.01*	0.03 ± 0.004

*F* female, *M* male

FEV_1_ – forced expiratory volume in one second

PD_20_ – the provocation dose of methacholine causing a 20 % decrease in FEV_1_

Data presented as Mean ± standard error of the mean

*p < 0.01 comparing with healthy subjects peripheral eosinophils count

^a^Geometric mean

### Study design

All subjects were informed about the requirements for participation in the study, informed written consent was obtained, inclusion/exclusion criteria were verified, and physical examination, spirometry, a methacholine challenge test, and a skin prick test were performed. Peripheral blood was taken if an individual met the criteria for the study. After extraction of eosinophils, for each study participant a separate co-culture with healthy ASMC was prepared. ASMC were cultured for 3 days in medium with serum and 1 day in serum-free medium with insulin-transferrin-selenium reagent, as described below, before co-culture. After 30, 45, 60, 120, and 240 min of co-culture, eosinophil adhesion was measured. After 24 h of co-culture, samples were collected for gene expression analysis. ASMC proliferation was measured after 48 and 72 h. Before the collection of samples for gene expression analysis and before ASMC proliferation measurements, all wells were incubated for 4 min with phosphate-buffered saline (PBS) containing 1 mM ethylenediaminetetraacetic acid (EDTA) until eosinophils detached from ASMC. This was done to avoid errors in gene expression and ASMC proliferation results caused by nonhomogeneous ASMC preparations. EDTA is a chelator of divalent ions than can suppress eosinophil adhesion through integrins by binding calcium ions. Because adhesion of eosinophils to ASMC is weaker than adhesion of ASMC to culture dishes, the incubation with PBS and EDTA allowed separation of these two types of cells. To confirm complete removal of eosinophils from ASMC, the cultures were observed through a microscope after all experiments. Samples for gene expression were lysed and cell proliferation was measured immediately after removal of EDTA from the culture to avoid adverse effects on cell viability.

### Lung function testing

Pulmonary function was tested using a pneumotachometric spirometer “CustovitM” (Custo Med, Germany). Baseline forced expiratory volume in one second (FEV_1_), forced vital capacity (FVC), and FEV_1_/FVC ratio were recorded as the highest of three reproducible measurements. The results were compared with the predicted values matched for age, body height, and sex according to the standard methodology.

### Measurement of airway responsiveness to methacholine

Airway responsiveness was assessed as changes in airway function after challenge with inhaled methacholine using a reservoir method. Methacholine was nebulized into a 10-L reservoir with a pressure nebulizer (Pari Provocation I; Pari, Stanberg, Germany). Aerolized methacholine was inhaled through a one-way valve at 5-min intervals starting with 15-μg methacholine dose and doubling it until a 20 % decrease in FEV_1_ from the baseline or the total cumulative dose of 3.87 mg was achieved. The bronchoconstricting effect of each dose of methacholine was expressed as a percentage of decrease in FEV_1_ from the baseline value. The provocative dose of methacholine causing a ≥ 20 % fall in FEV_1_ (PD_20_) was calculated from the log dose–response curve by linear interpolation of two adjacent data points.

### Skin prick testing

All patients were screened for allergy by the skin prick test using standardized allergen extracts (Stallergenes S.A., France) for the following allergens: *D. pteronyssinus*, *D. farinae*, cat and dog dandruff, 5 mixed grass pollen, birch pollen, mugwort, *Alternaria*, *Aspergillus* and *Cladosporium*. Temoin was used for a negative control, and histamine hydrochloride (10 mg/mL) was used for a positive control. Skin testing was read 15 min after application. The results of skin prick test were considered positive if the mean wheal diameter was ≥ 3 mm.

### Analysis of peripheral blood cells

The analysis of peripheral blood cells was performed on an automated hematology analyzer XE-5000™ (Sysmex, Japan).

### Sputum induction, processing and cells analysis

After baseline FEV_1_ and FVC measurements, subjects inhaled 10 mL of sterile hypertonic saline solution (3 %, 4 %, or 5 % NaCl, Ivex Pharmaceuticals, USA) at room temperature. Nebulised solution was given for three periods of 5 min at most by an ultrasonic nebuliser ULTRA-NEB™ (DeVilbiss, USA). The subjects were instructed to cough sputum into containers. If any symptoms occurred, nebulisation was discontinued. It was stopped after expectoration of an adequate amount of sputum. In order to detect a possible decrease in FEV_1_, spirometry was performed after each inhalation. Sputum was poured into a Petri dish and separated from saliva. A 4-fold volume of freshly prepared 0.1 % dithiothreitol (DTT; Sigma-Aldrich) was added. The mixture was vortexed and placed on ice and rocked on an orbital shaker OS-10 (BIOSAN, Latvia) for 15 min. Next, an equal volume of phosphate-buffered saline solution (PBS; Sigma-Aldrich) was added to DTT. The cell pellet was separated using a 40-μm cell stainer (Becton Dickinson, USA). The mixture was centrifuged for 10 min at 4 °C; the supernatant was aspirated and stored at −70 °C for later analysis. The total cell counts, percentage of epithelial cells, and cell viability were investigated using a Neubauer hemocytometer (Heinz-Herenz, Germany) under a microscope (BX43, OLYMPUS, USA) by employing the Trypan blue exclusion method. The cytospin samples of induced sputum were prepared using a cytofuge instrument (Shandon Southern Instruments, USA). Supernatant with and without DTT was stored in 2 mL microtubes at −70 °C for further researches. The prepared sputum cytospins were stained by the May-Grünwald-Giemsa method for differential cell counts. Cell differentiation was determined by counting approximately 400 cells in random fields of view under a light microscope, excluding squamous epithelial cells. The cells were identified by standard morphological criteria, nuclear morphology, and cytoplasmic granulation.

### Peripheral blood collection and isolation of eosinophils

Peripheral blood samples for eosinophil isolation were collected into sterile Vacutainer tubes with EDTA. Polymorphonuclear leukocytes (PMN) were isolated by high-density gradient centrifugation. The whole blood was layered on Ficoll-Paque PLUS (GE Healthcare, Finland) and centrifuged at 1000 × *g* for 30 min at room temperature, and then PMN were separated by hypotonic lysis of erythrocytes. Eosinophils were separated using a magnetic eosinophil isolation kit (Miltenyi Biotec, USA). MACS buffer (containing 0.5 % bovine serum albumin (BSA) and 2 mM EDTA in PBS, pH 7.2) was prepared by diluting MACS BSA Stock Solution 1:20 in autoMACS Rinsing Solution. The granulocyte pellet was resuspended in cold MACS buffer (40 μL per 1 × 10^7^ cells) and incubated with Biotin-Antibody Cocktail (biotin-conjugated monoclonal antibodies against CD2, CD14, CD16, CD19, CD56, CD123, and CD235a (glycophorin A)) (10 μL per 1 × 10^7^ cells) for 10 min. After incubation, 20 μL of Anti-Biotin MicroBeads (microbeads conjugated to monoclonal mouse anti-biotin IgG1) per 1 × 10^7^ cells was added, mixed, and incubated for an additional 15 min at 4 °C. An LS column (Miltenyi Biotec, USA) was prepared during this time by placing the column in the magnetic field of a MACS Separator and washing it with 2 mL of MACS buffer. A pre-separation filter (30 μm; Miltenyi Biotec) was rinsed with MACS buffer and placed on top of the column. The cells were then applied to the pre-separation filter/LS column, and the magnetically labeled non-eosinophils were retained on the column in the magnetic field of the Separator while the unlabeled eosinophils passed through the column. Cells were eluted with 5 mL of MACS buffer and centrifuged (400 × *g*, 10 min, 4 °C), and the pellet was resuspended in 1 mL of Dulbecco’s modified Eagle’s medium (DMEM) (Biological Industries, Israel) to a final concentration of 2 × 10^6^ cells/mL. Eosinophils were counted using an ADAM automatic cell counter (Witec AG, Switzerland). Blood eosinophil viability was assessed by Trypan Blue exclusion and found to be at least 98 %. To check eosinophil purity after magnetic separation, a diluted eosinophil suspension was fixed with methanol on a glass plate, stained with May–Grunwald–Giemsa dyes, and inspected by light microscopy to determine whether there were any neutrophils in the suspension.

### Airway smooth muscle cell culture

Healthy human ASMC isolated from one donor and immortalized by stable expression of hTERT were used for the experiments [19]. Cells were maintained in 75 cm^2^ Falcon culture flasks under standard culture conditions of 5 % CO_2_ in air at 37 °C with medium renewal every 2–3 days. For all experiments, passaged cells were grown on plastic dishes in DMEM (GIBCO® by Life Technologies, Paisley, UK) supplemented with streptomycin/penicillin (2 % v/v; Pen-Strep, GIBCO® by Life Technologies, Paisley, UK), amphotericin B (1 % v/v; GIBCO® by Life Technologies), and fetal bovine serum (10 % v/v; GIBCO® by Life Technologies). Cells were transferred and medium was refreshed every 48–72 h. After reaching sufficient confluency, cells were passaged by trypsinization. Cells were serum deprived in DMEM supplemented with antibiotics and insulin, transferrin, and selenium reagent (GIBCO® by Life Technologies) before each experiment to stop cell proliferation and avoid possible errors in proliferation and gene expression measurements due to the effect of serum in the growth medium.

### Co-culture of airway smooth muscle cells and eosinophils

**Fig. 1:**
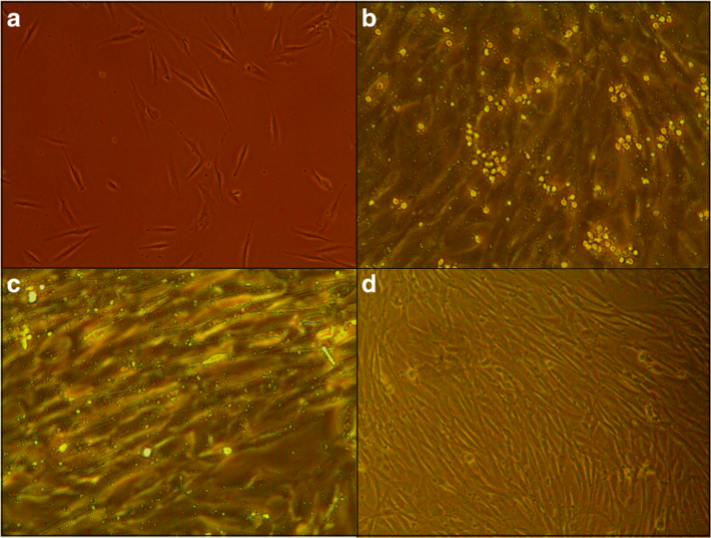
Combined culture of airway smooth muscle cells (ASMC) and eosinophils. **a** ASMC incubated alone on the first day of cultivation. **b** Combined culture of confluent ASMC and eosinophils from asthmatic subjects after 30 min of incubation, demonstrating the interaction of ASMC with eosinophils. **c** Combined culture of confluent ASMC with eosinophils from asthmatic individuals after 30 min of incubation and three washes with warm phosphate-buffered saline (PBS), demonstrating the proportion of eosinophils stably adhering to viable ASMC. **d** ASMC after 4 min of incubation with warm PBS containing 1 mM EDTA, demonstrating effective separation of combined cultures. Sufficient confluency was considered to be 75 %, equivalent to 2 × 10^5^ ASMC. Eosinophils from asthmatic subjects were seeded at 2 × 10^5^ cells per well. Magnification in panels A and D: wide-field eyepiece, ×10; objective, ×10. Magnification in panels B and C: wide-field eyepiece, ×20; objective, ×10

For all experiments, individual co-cultures of eosinophils and human bronchial smooth muscle cells were prepared. We used 0.5 × 10^5^ viable eosinophils and 2 × 10^5^ ASMC for all experiments except the one shown in Fig. 1, in which 2 × 10^5^ viable eosinophils were used. For cell visualization, we used an inverted microscope (CETI Inverso TC100, Medline Scientific, UK) with a 10×/22 mm wide-field eyepiece and PH 10×/0.25 objective and an installed CETI Si-5C camera (Si-Cap software, Version 2.1, 2012).

### Eosinophil adhesion assay

**Fig. 2:**
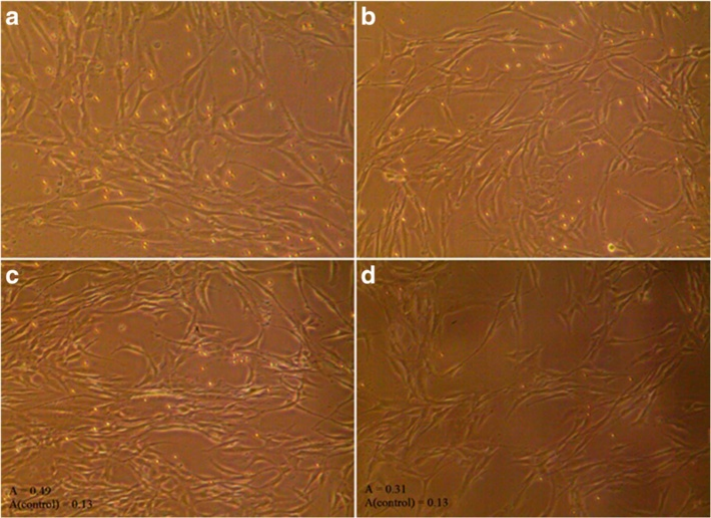
Combined culture of airway smooth muscle cells (ASMC) and eosinophils from asthmatic and healthy individuals. **a** ASMC incubated with eosinophils from asthmatic subjects. **b** Combined culture of ASMC and eosinophils from healthy subjects. **c** Combined culture of ASMC and eosinophils from asthmatic subjects after 30 min of incubation and three washes with warm phosphate-buffered saline (PBS). **d** ASMC after 30 min of co-culture with eosinophils from healthy subjects and three washes with warm PBS. The number of eosinophils (0.5 × 10^5^) and ASMC (2 × 10^5^) per well is the same in all panels. A: absorbance at 490 nm, representing eosinophil peroxidase activity in ASMC and eosinophil co-cultures. A(control): absorbance in control ASMC cultured without eosinophils. Sufficient confluency was considered to be 75 %, equivalent to 2 × 10^5^ cells. Magnification: wide-field eyepiece, ×10; objective, ×10

ASMC were seeded in 6-well plates (1 × 10^4^ per well) and grown in DMEM for 3 days in 5 % CO_2_ at 37 °C. Then, the medium was removed and wells were washed twice with PBS. DMEM without fetal bovine serum but supplemented with 10 % insulin-transferrin-selenium reagent was added, and cells were incubated for 1 day. Next, a co-culture with eosinophils was prepared. Eosinophils were allowed to adhere to ASMC for 30, 45, 60, 120, and 240 min. After incubation, non-adhered cells were removed and the remaining cells were washed twice with PBS. Eosinophil adhesion was determined by measuring residual eosinophil peroxidase (EPO) activity of adherent cells as previously described [20]. Because intercellular EPO levels are decreased in eosinophils from asthmatic individuals owing to degranulation [21], increased EPO activity can be determined for higher numbers of eosinophils stably adhering to the ASMC surface. In Fig. 2 we provide visual confirmation of an increased number of asthma-associated eosinophils stably adhering to ASMC. To assay EPO activity, 462 μL of RPMI-1640 medium without phenol red and 462 μL of EPO substrate (1 mM H_2_O_2_, 1 mM *o*-phenylenediamine, and 0.1 % Triton X-100 in Tris buffer, pH 8.0) were added to each well. After 30 min at room temperature, 231 μL of 4 M H_2_SO_4_ was added to each well to stop the reaction and the absorbance was measured at 490 nm in a microplate reader. Results are expressed relative to substrate oxidation levels in control cells (ASMC cultured without eosinophils). The absorbance in control cells is presented as 100 % and eosinophil adhesion is expressed as the difference from the control value.

### Alamar blue proliferation assay

Cells were grown in 6-well cluster plates. ASMC proliferation was assessed as described previously. Healthy ASMC were co-cultured with eosinophils isolated from asthma patients and healthy individuals for 48 and 72 h. Thereafter, all cells were washed once with warm PBS and incubated for 4 min with warm PBS supplemented with 1 mM EDTA to detach eosinophils from ASMC. Proliferation was evaluated by incubating wells with Hank’s Balanced Salt Solution containing Alamar blue solution (10 % v/v; Invitrogen™ by Life Technologies, Paisley, UK). Conversion of Alamar blue to the reduced form was dependent on the metabolic activity of ASMC and was assayed by dual-wavelength spectrophotometry at wavelengths of 570 nm and 600 nm. As indicated by the manufacturer, the degree of Alamar blue conversion is proportional to the number of viable cells. The data are expressed as the percent increase or decrease in Alamar blue conversion by ASMC compared with control cells (ASMC cultured without eosinophils), which did not proliferate during the co-culturing period.

### RNA isolation and real-time PCR

ASMC co-cultured with eosinophils and control ASMC (cultured under the same conditions but without eosinophils) were prepared for total RNA extraction by washing the cells with warm PBS and incubating them for 4 min with warm PBS supplemented with 1 mM EDTA to detach eosinophils. Total RNA was extracted using the miRNeasy Mini Kit (QIAGEN, USA) according to the manufacturer’s instructions. Then, real-time PCR was performed using the Power SYBR® Green RNA-to-C_T_™ 1-Step Kit (Applied Biosystems, USA) in a 7500 Fast Real-Time PCR System, as follows: reverse transcription at 48 °C for 30 min; activation of AmpliTaq Gold® DNA Polymerase, UP (Ultra-Pure) at 95 °C for 10 min; and 40 cycles of denaturation at 95 °C for 15 s and annealing and extension at 60 °C for 1 min. Real-time PCR data were analyzed using the comparative cycle threshold method (the amount of target gene was normalized to the endogenous reference gene 18S ribosomal RNA). A difference of 1 C_q_ (ΔC_q_ = 1, where C_q_ is the amplification cycle number) after normalization to the reference gene indicates a twofold higher expression level of the investigated gene. Relative differences were determined by normalization of test sample ΔC_q_ values to control sample ΔC_q_ values with the equation ΔC_q_(control) − ΔC_q_(test). The following primers were used to analyze gene expression: Wnt-5a, Fwd 5′-GGG TGG GAA CCA AGA AAA AT-3′ and Rev 5′-TGG AAC CTA CCC ATC CCA TA-3′; TGF-β_1_, Fwd 5′-GTA CCT GAA CCC GTG TTG CT-3′ and Rev 5′-GAA CCC GTT GAT GTC CAC TT-3′; 18S rRNA, Fwd 5′-CGC CGC TAG AGG TGA AAT TC-3′ and Rev 5′-TTG GCA AAT GCT TTC GCT C-3′; collagen type Iα1, Fwd 5′-AGC CAG CAG ATC GAG AAC AT-3′ and Rev 5′-TCT TGT CCT TGG GGT TCT TG-3′; and fibronectin, Fwd 5′-TCG AGG AGG AAA TTC CAA TG-3′ and Rev 5′-ACA CAC GTG CAC CTC ATC AT-3′.

### Statistical analysis

Statistical analysis was performed with GraphPad Prism 6 for Windows (Version 6.05, 2014; GraphPad Software, Inc., San Diego, CA, USA). Normally distributed data are presented as mean and standard error of the mean (SEM). Non-normally distributed data are expressed as median and range and were analyzed using nonparametric tests. Significant differences between two independent groups were determined by the Mann–Whitney *U* test. The Wilcoxon matched-pairs signed rank test was used for dependent groups. Statistical significance was assumed at a *p* value of <0.05.

## Results

### Characteristics of the studied subjects

Eighteen nonsmoking adults participated in this study: 8 steroid-free patients with intermittent or mild-to-moderate persistent asthma, and 10 healthy subjects, who constituted the control group. The demographic, clinical, and laboratory data of the study subjects are presented in Table 1. There were no significant age or sex differences between the groups. Lung function was similar in both groups, whereas airway hyperresponsiveness and skin prick positivity were detected only in the asthma group. The number of eosinophils was significantly higher in peripheral blood and induced sputum from patients with asthma compared with healthy individuals.

### Eosinophil adhesion to airway smooth muscle cells

**Fig. 3:**
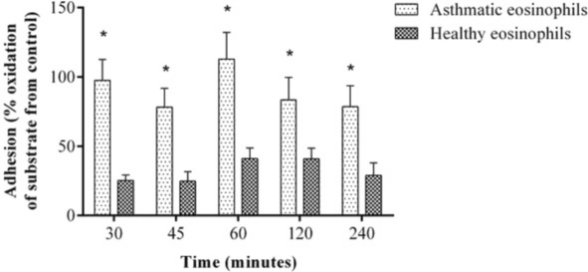
Eosinophil adhesion to airway smooth muscle cells at various time points. Results are shown as mean ± SEM. Asthma patients, *n* = 8; healthy subjects, *n* = 10. *p < 0.05 compared with healthy individuals. Eosinophil peroxidase substrate oxidation is expressed as the percent difference from the absorbance in control ASMC cultured without eosinophils

Adhesion was expressed as EPO substrate oxidation (absorbance at 490 nm) in co-cultures of eosinophils and ASMC relative to EPO substrate oxidation in control ASMC (cultured without eosinophils); the control value is set as 100 %, and eosinophil expression is the difference from the control value. Adhesion of eosinophils from asthma patients was significantly greater than adhesion of eosinophils from healthy subjects at all time points: 30 min (97.5 ± 15.2 % vs 25.3 ± 4.1 %; *p* < 0.05), 45 min (78.2 ± 13.6 % vs 24.8 ± 6.9 %; *p* < 0.05), 60 min (112.9 ± 19.2 % vs 41.1 ± 7.7 %; *p* < 0.05), 120 min (83.4 ± 16.3 % vs 40.9 ± 7.8 %; *p* < 0.05), and 240 min (78.5 ± 15.2 % vs 29.0 ± 9.0 %; *p* < 0.05). Moreover, the adhesion of eosinophils to ASMC did not differ among time points within the asthma patients group or within the healthy subjects group (Fig. 3).

### Effect of eosinophils on expression of Wnt-5a and TGF-β_1_ genes in airway smooth muscle cells

**Fig. 4:**
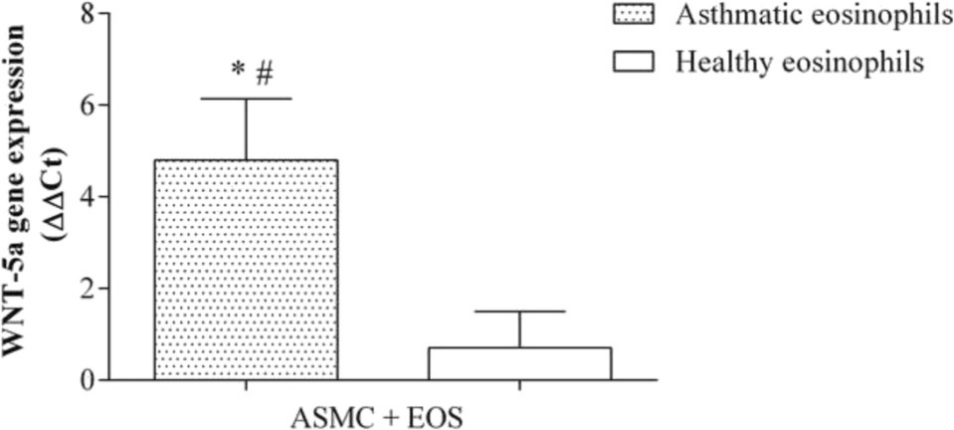
Effect of co-culture of airway smooth muscle cells (ASMC) with eosinophils on Wnt-5a gene expression. Gene expression was measured after 24 h of incubation. Results are presented as mean − ΔΔC_t_ ± SEM. Asthma patients, *n* = 8; healthy subjects, *n* = 10. *p < 0.05 compared with eosinophils from healthy subjects; #*p* < 0.05 compared with control ASMC cultured without eosinophils

**Fig. 5:**
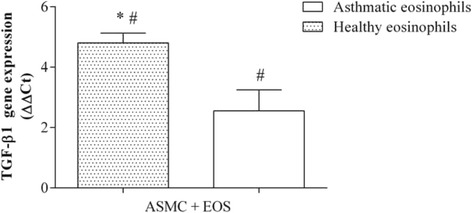
Effect of co-culture of airway smooth muscle cells (ASMC) with eosinophils on TGF-β_1_ gene expression. Gene expression was measured after 24 h of incubation. Results are presented as mean − ΔΔC_t_ ± SEM. Asthma patients, *n* = 8; healthy subjects, *n* = 10. *p < 0.05 compared with eosinophils from healthy subjects; #*p* < 0.05 compared with control ASMC cultured without eosinophils

After 24 h of co-culture with eosinophils from asthmatic subjects (0.5 × 10^5^ eosinophils and 2 × 10^5^ ASMC), Wnt-5a gene expression in ASMC increased 4.80 ± 1.34 fold (mean ± SEM; *p* < 0.05) compared with control ASMC cultured without eosinophils (Fig. 4). No significant effect on Wnt-5a gene expression was detected after incubation of ASMC with eosinophils from healthy subjects. Furthermore, eosinophils from both asthmatic and healthy subjects induced overexpression of the TGF-β_1_ gene in ASMC, but the effect of eosinophils from asthmatic subjects was significantly greater (4.80 ± 0.33 fold vs 2.56 ± 0.69 fold; mean ± SEM; *p* < 0.05) (Fig. 5).

### Effect of eosinophils on expression of collagen and fibronectin genes in airway smooth muscle cells

**Fig. 6:**
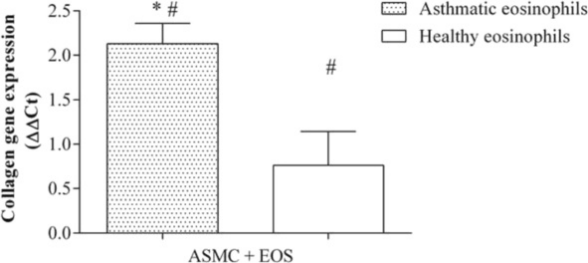
Effect of co-culture of airway smooth muscle cells (ASMC) with eosinophils on collagen gene expression. Gene expression was measured after 24 h of incubation. Results are presented as mean − ΔΔC_t_ ± SEM. Asthma patients, *n* = 8; healthy subjects, *n* = 10. *p < 0.05 compared with eosinophils from healthy subjects; #*p* < 0.05 compared with control ASMC cultured without eosinophils

**Fig. 7:**
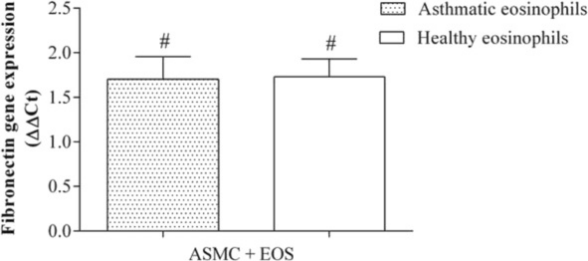
Effect of co-culture of airway smooth muscle cells (ASMC) with eosinophils on fibronectin gene expression. Gene expression was measured after 24 h of incubation. Results are presented as mean − ΔΔC_t_ ± SEM. Asthma patients, *n* = 8; healthy subjects, *n* = 10. #*p* < 0.05 compared with control ASMC cultured without eosinophils

After 24 h of co-culture with eosinophils from asthma patients (0.5 × 10^5^ eosinophils and 2 × 10^5^ ASMC), collagen gene expression in ASMC increased 2.13 ± 0.23 fold (mean ± SEM) compared with control ASMC cultured without eosinophils. No significant effect on collagen gene expression was detected after incubation of ASMC with eosinophils from healthy subjects (Fig. 6). Moreover, eosinophils from both asthmatic and healthy subjects significantly increased fibronectin gene expression in ASMC compared with control ASMC cultured without eosinophils (1.71 ± 0.25 fold and 1.73 ± 0.20 fold, respectively; mean ± SEM; *p* < 0.05) (Fig. 7).

### Proliferation of airway smooth muscle cells after co-culture with eosinophils

**Fig. 8:**
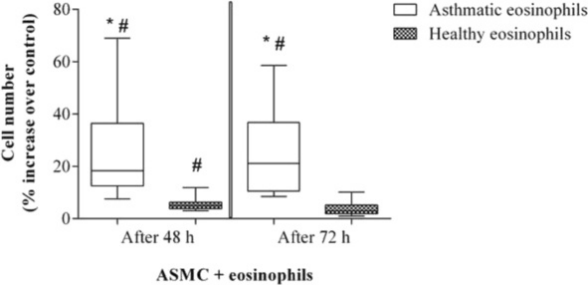
Airway smooth muscle cell (ASMC) proliferation after co-culture with eosinophils from asthmatic and healthy subjects. Results are shown as median (ranges). Asthma patients, *n* = 8; healthy subjects, *n* = 10. *p < 0.05 compared with eosinophils from healthy subjects; #*p* < 0.05 compared with control ASMC cultured without eosinophils. Proliferation is expressed as the percent increase in Alamar blue reagent reduction compared with control ASMC cultured without eosinophils

ASMC proliferation was measured after 48 and 72 h of incubation with eosinophils. Proliferation results were expressed as the percent increase in Alamar blue reagent reduction over the control, indicating an increase in the number of viable cells. Eosinophils from asthma patients significantly increased ASMC proliferation after 48 h (18.4 %, range 7.6–69.0 %, vs 5.0 %, range 3.0–11.9 %; *p* < 0.05) and 72 h of co-culture (21.2 %, range 8.5–58.6 %, vs 3.0 %, range 1.1–10.2 %; *p* < 0.05) (Fig. 8).

## Discussion

This study showed that eosinophils from peripheral blood of steroid-free asthma patients and healthy individuals can adhere to ASMC, enhance TGF-β_1_ and ECM protein (fibronectin and collagen) gene expression in ASMC, and increase ASMC proliferation in vitro. These effects were significantly more pronounced after co-culture of ASMC with eosinophils from asthmatic subjects versus eosinophils from healthy subjects. Furthermore, eosinophils from asthmatic subjects enhanced Wnt-5a gene expression in ASMC, whereas eosinophils from healthy subjects did not affect the expression of this gene.

Eosinophils are circulating granulocytes that are considered to be the major inflammatory cells in asthma pathogenesis, with a major role in the development of airway remodeling [22]. In asthma, increased airway smooth muscle mass occurs through both cellular hypertrophy and hyperplasia [23]. Normally, eosinophils are not detectable or their amount is minimal in the bronchial submucosa or bronchial secretions, whereas in asthma, eosinophilic inflammation is often presented, with a strong predominance in the submucosa close to ASMC [24].

Infiltration of eosinophils into the airway is regulated by various chemokines and cytokines, such as eotaxin, RANTES, IL-5, and IL-13, and involves signaling via Rho-kinase [25, 26]. Hughes et al. demonstrated that eosinophils can interact with ASMC directly through an integrin-ligand interaction [27]. To investigate eosinophil adhesion to ASMC, we measured eosinophil peroxidase activity by monitoring the oxidation of *o*-phenylenediamine in vitro. This is a reliable method for the determination of eosinophil numbers [20]. We have shown that increased eosinophil peroxidase activity correlates with increased numbers of eosinophils stably adhered to ASMC (Fig. 2). We found that eosinophils adhere to ASMC rapidly and continuously during incubation periods of 30 to 240 min. These results suggest that eosinophils may adhere to ASMC quickly after they migrate into the airway. Eosinophils isolated from asthma patients had significantly increased adhesive activity compared with eosinophils isolated from healthy individuals. IL-5 regulates eosinophil functions (development, activation, migration, survival) and, together with eotaxin, is a critical molecular switch for the induction of blood and tissue eosinophilia [28, 29]. Eosinophils isolated from asthmatic individuals may be more active in vivo than those isolated from non-asthmatic individuals, leading to enhanced eosinophil adhesion to ASMC because of increased levels of eotaxin and RANTES in asthma [30, 31].

Eosinophils are the main inflammatory cells releasing TGF-β_1_ in asthma [32]. A recent study revealed that TGF-β_1_ may affect noncanonical Wnt signaling by inducing the expression of Wnt-5a in ASMC, where it can mediate the expression of ECM proteins [12]. To investigate the effect of eosinophils from asthma patients on Wnt-5a and TGF-β_1_ gene expression, we determined the total mRNA levels of these genes in ASMC after incubation with eosinophils. Only eosinophils isolated from asthma patients significantly increased Wnt-5a gene expression, while eosinophils from both asthmatic and non-asthmatic individuals enhanced TGF-β_1_ gene expression, although the increase in TGF-β_1_ gene expression was significantly greater with eosinophils from asthma patients. To our knowledge, these are the first data describing the effect of eosinophils from asthmatic individuals on Wnt activation in human airway smooth muscle cells. We suggest that while eosinophils from both asthmatic and healthy individuals increase TGF-β_1_ expression in ASMC, the amount of TGF-β_1_ in ASMC after co-culture with eosinophils from healthy subjects is not sufficient to activate noncanonical Wnt signaling. Eosinophils from patients with asthma have much greater activity and more efficaciously enhance the production of TGF-β_1_ in ASMC, which in turn may affect Wnt-5a gene expression. Moreover, the eosinophil is a TGF-β_1_-producing cell, and eosinophil-derived TGF-β_1_ may also affect noncanonical Wnt signaling. Noncanonical Wnt signaling pathway activation is associated with cell migration and polarity [33, 34]. To evaluate the effect of crosstalk between TGF-β_1_ and noncanonical Wnt signaling on ECM protein expression, we determined the expression of collagen type Iα1 and fibronectin genes in ASMC after exposure to eosinophils. Only exposure to peripheral blood eosinophils from asthma patients significantly increased collagen gene expression in ASMC, while eosinophils from both asthmatic and healthy individuals increased fibronectin gene expression, compared with control ASMC cultured without eosinophils. These results suggest that eosinophils from healthy subjects may promote expression of fibronectin, one of the main components of ECM, by ASMC; therefore, airway remodeling in asthma may be associated with increased expression of another ECM protein: collagen. Moreover, our results suggest that airway remodeling during asthma may be related to increased noncanonical Wnt signaling pathway activity due to altered eosinophil adhesion.

Direct contact of eosinophils with ASMC induces the secretion of TGF-β_1_ from ASMC. Furthermore, TGF-β_1_ secretion is related to increased ECM protein production, leading to enhanced proliferation in vitro [12]. In our study, healthy human ASMC were co-cultured with eosinophils from asthmatic or healthy subjects to investigate how eosinophils promote alterations in ASMC homeostasis in asthma. Noncanonical Wnt-5a signaling is activated by and necessary for TGF-β_1_-induced ECM production by ASMC [12], and ECM proteins can modulate asthmatic ASMC proliferation via an autocrine and paracrine mechanism [13]. We hypothesized that greater proliferation of these cells in asthma could be associated with a TGF-β_1_-promoted disorder in noncanonical Wnt signaling as well as increased ECM protein production. To investigate the effect of eosinophils on ASMC proliferation, we co-cultured ASMC with eosinophils from asthma patients or healthy individuals and measured cell numbers after 48 and 72 h. We found that eosinophils from asthma patients increased ASMC proliferation more significantly than eosinophils from healthy individuals did. We speculate that this may be because of increased Wnt-5a gene expression after co-culture with eosinophils from asthmatic individuals, as discussed above. This supposition is supported by findings that Wnt-5a expression is increased in ASMC from asthma patients [12] and that Wnt-5a gene expression is enriched in biopsies from patients with Th2-high asthma [35].

A possible limitation of this study is that we used peripheral blood eosinophils instead of airway tissue eosinophils. Normally, the eosinophils affecting ASMC have migrated from peripheral blood into the airway [36]. Recent studies have demonstrated that eosinophil transmigration is influenced by cytokines, chemokines, and other locally produced soluble mediators that control interactions between adhesion molecules on the surface of eosinophils and their counter-structures on opposing cells or matrix proteins [7]. Nevertheless, our study model is appropriate for investigations in vitro because eosinophils from asthmatic individuals are known to be primed or activated in vivo and show enhanced responses to different stimuli in vitro [37].

## Conclusions

In summary, eosinophils from patients with asthma increase Wnt-5a and TGF-β_1_ gene expression in ASMC, enhance the expression of collagen and fibronectin, and promote ASMC proliferation. We propose that these effects are mechanistically linked and thus, in the presence of eosinophilic airway inflammation, these cells can contribute to airway remodeling through ASMC proliferation by activating the noncanonical Wnt signaling pathway.

## Abbreviations

ASMC, airway smooth muscle cells; ECM, extracellular matrix; TGF-β_1_, transforming growth factor-β_1_; Wnt, wingless/integrase-1 signaling; Wnt-5a, noncanonical Wnt signaling pathway activating ligand 5a; WNT-5a, gene, coding Wnt-5a ligand; PCR, polymerase chain reaction; EDTA, ethylenediaminetetraacetic acid; FEV_1_, forced expiratory volume in one second; FVC, forced vital capacity; PBS, phosphate-buffered saline, DTT, dithiothreitol; EPO, eosinophil peroxidase.
